# Real-world outcomes after concurrent chemo-radiotherapy in patients with locally advanced esophageal and gastroesophageal junction cancer

**DOI:** 10.2340/1651-226X.2025.44013

**Published:** 2025-10-15

**Authors:** Hanna Rahbek Mortensen, Lone Hoffmann, Marianne Nordsmark, Lise Bech Jellesmark Thorsen, Ditte Sloth Møller

**Affiliations:** a Danish Center of Particle Therapy, Aarhus University Hospital, Aarhus, Denmark; b Department of Clinical Medicine, Faculty of Health Science, Aarhus University, Aarhus, Denmark; c Department of Oncology, Aarhus University Hospital, Aarhus, Denmark

**Keywords:** Esophageal cancer, radiotherapy, outcome, morbidity, real-world data

## Abstract

**Background and purpose:**

Standard treatment for esophageal (EC) and gastroesophageal junction (GEJ) cancer includes neoadjuvant chemo-radiotherapy (nCRT), followed by surgery or definitive chemo-radiotherapy (dCRT) for inoperable patients. This study assessed real-world survival and morbidity in EC patients treated with radiotherapy (RT).

**Patient/material and methods:**

In this retrospective study, 417 patients with EC or GEJ cancer received nCRT or dCRT between 2012 and 2021 at a single center. We evaluated overall survival (OS), loco-regional control, progression-free survival, failure patterns, and toxicity.

Data were sourced from clinical and treatment records. Patients were treated following national guidelines and received intensity-modulated radiotherapy and daily cone-beam Computed Tomography (CT) for setup. Radiotherapy doses were 41.4–66 Gy in 23–33 fractions.

**Results:**

Of the patients, 250 received nCRT, and 167 received dCRT. Most (86%) had T3-T4 tumors, and 65% had node-positive disease. Histologies were adenocarcinoma (50%) and squamous cell carcinoma (45%). A total of 88% completed RT, and 92.4% of nCRT patients proceeded to surgery. Median OS was 31 months for nCRT and 24 months for dCRT; 3-year OS was 46% and 38%, respectively. Disease recurrence occurred in 46% with a median interval of 20 months. Multivariable analysis identified OS-associated factors for both nCRT and dCRT. Acute toxicity was common but generally mild; late side effects were not systematically recorded.

**Interpretation:**

In clinical practice, OS after nCRT or dCRT was as expected. Most patients undergoing nCRT proceeded to surgery. Toxicity was frequent but manageable.

## Introduction

Carcinoma of the esophagus and gastroesophageal junction (GEJ) is the 8th most common cancer and the 6th most common cause of cancer death [1]. In Denmark, approx. 90 new cases are diagnosed per year, with a 5-year survival of less than 20% for the entire patient group and a 5-year survival of approx. 40% for the curatively treated patients [2, 3].

Until 2025, standard curative treatment for patients with resectable esophageal (EC) and GEJ cancer was perioperative chemotherapy [4] or neoadjuvant chemo-radiotherapy (nCRT) followed by surgery [5] and adjuvant immunotherapy for patients without pathological complete response (pCR) [6]. nCRT demonstrated a beneficial effect on survival compared with surgery alone in the CROSS trial, randomizing patients to either surgery alone or the CROSS regimen with nCRT consisting of 41.4 Gy in 23 fractions combined with weekly courses of carboplatin and paclitaxel followed by surgery [5, 7]. Other standard regimens prescribe a total radiation dose ranging between 40.0 Gy and 50.4 Gy in 1.8 to 2 Gy fractions and/or concurrent cisplatin plus 5-fluouracil-based chemotherapy [8].

Lately, the question of perioperative chemotherapy for adenocarcinoma has been examined in the Neo-AEGIS and ESOPEC trials [9, 10]. The Neo-AEGIS study compared CROSS versus MAGIC or FLOT, all except CROSS only considering adenocarcinoma. The MAGIC regimen consists of perioperative chemotherapy with epirubicin plus cisplatin or oxaliplatin plus fluorouracil or capecitabine, and the FLOT regimen consists of perioperative chemotherapy with fluorouracil, leucovorin, oxaliplatin, and docetaxel. An interim analysis of the Neo-AEGIS study showed no difference in overall survival (OS) as well as no major differences in surgery-related complications and health-related quality of life outcomes [9]. The ESOPEC study, a German multicenter randomized phase III trial, compared nCRT versus perioperative FLOT for primary EC and GEJ adenocarcinomas stage cT1cN+, cT2-4a cN+, or cT2-41 cN0 disease without metastatic spread. This study showed that perioperative chemotherapy with FLOT plus surgery was superior to the CROSS regimen, and consequently, FLOT became the new standard of care in EC and GEJ adenocarcinomas [10]. The outcome of the CROSS arm in ESOPEC was considerably lower than results from previous trials and real-world data, which could be due to poor radiotherapy (RT) quality; however, data on RT treatment details and quality assurance are sparse.

Patients with locally advanced EC or GEJ cancer, who do not want or are not candidates for surgery, will receive definitive chemo-radiotherapy (dCRT). dCRT as an organ preserving strategy for patients with locally advanced EC squamous cell carcinoma (SCC) is used with increasing frequency and is currently investigated in the Dutch SANO trial [11] and the international multicenter NEEDs trial [12]. The ARTDECO trial studied the effect of RT dose escalation and established 50–50.4 Gy in 25–28 fractions, 5 fractions per week, combined with weekly courses of carboplatin and paclitaxel as standard of care in dCRT [13].

Thus, clinical practice is changing, and in the light of this, the aim of the current study was to examine real-world outcomes in EC and GEJ patients treated with (chemo-)RT with curative intent.

## Patients/material and methods

### Patients

Between 2012 and 2021, consecutive patients with locally advanced EC and GEJ cancer, planned for curatively intended concomitant chemo-radiotherapy with or without subsequent surgery, were identified at the ECV center at Aarhus University Hospital, Denmark.

All patients were diagnosed based on Positron Emission Tomography-Computed Tomography (PET-CT), gastroscopy, biopsy, and lung function examination and discussed at a multidisciplinary team (MDT) conference advising treatment recommendations. Cardiac evaluation and kidney function tests were performed before treatment started.

Follow-up included clinical examination at baseline, weekly during treatment, and at the end of treatment for all patients. After completing nCRT and surgery, patients were followed at the Department of Surgery (section for upper gastrointestinal surgery) for 3 years. Patients treated with dCRT were followed at the Department of Oncology for 5 years. As standard of care, all patients with suspected recurrence and performance status (PS) 0–2 were referred to the ECV center at Aarhus University Hospital for diagnostic assessment and treatment. Data on local, regional, and distant recurrence as well as death were obtained from the electronic patient files.

### Radiotherapy

**Figure 1:**
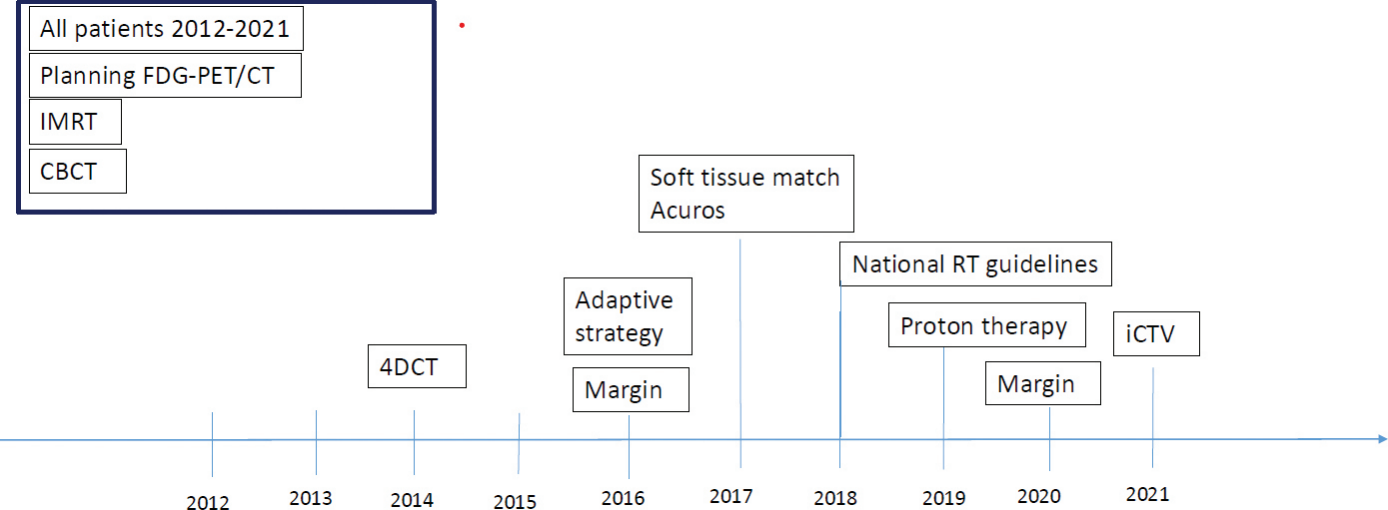
Change in treatment planning in the study period. FDG-PET/CT: Fluorodeoxyglucose – Positron Emission Tomography/Computed Tomography; IMRT: Intensity-Modulated Radiotherapy; CBCT: Cone-Beam Computed Tomography; 4DCT: 4 dimensional Computed Tomography; iCTV: Internal Clinical Target Volume.

During the 10-year period from 2012 to 2021, RT planning and treatment delivery were technically improved, as visualized in Figure 1. Ahead of this period, planning fluorodeoxyglucose (FDG)-PET/CT, Intensity-Modulated Radiotherapy (IMRT) planning, and daily cone-beam CT (CBCT) imaging for treatment guidance were implemented as standard procedures.

Patients were immobilized in a custom-made vacuum pillow. Target delineation and treatment planning were based on the mid-ventilation phase of a 4-dimensional PET-CT scan. Gross tumor volume (GTV) for both primary tumor and loco-regional malignant lymph nodes were delineated and discussed with a radiology specialist and a nuclear medicine specialist using all available information from the diagnostic PET-CT and the subsequent planning PET-CT. For the primary tumor, a standard clinical target volume (CTV) margin of 3 cm cranio-caudally and 1 cm radially was used except for tumors in the most cranial and caudal parts, where a 2 cm margin was allowed. For malignant lymph nodes, a CTV margin of 1 cm in all directions was used, and elective irradiation was only used for cervical tumors. Consensus Danish national guidelines were developed in 2018 (Supplementary 1).

From 2012 to 2016, the planning target volume (PTV) was defined by adding a generic margin to the CTV of 5 mm in the transversal and 8 mm in the cranio-caudal directions. The margins were changed in 2016 after collecting institutional numbers on patient setup uncertainties and implementing adaptive RT [14] to 8 mm transversally and 11 mm cranio-caudally. Finally, based on a study on implanted markers, the margins were changed in 2020 to 7mm transversally and 9 mm cranio-caudally for targets cranial to the base of the heart and 9 mm left-right, 11 mm anterior-posterior, and 9 mm cranio-caudally for targets caudal to the base of the heart [15]. For the latter margins, the PTV was generated based on an iCTV accounting for the respiratory motion [16].

Treatment planning was performed in Eclipse versions 7.6–15.6 (Varian Medical Systems Palo Alto, CA), using IMRT technique. The total dose was prescribed as the mean dose to the PTV, and the PTV was enclosed by the 95% isodose surface. The prescribed radiation dose ranged from 41.4 to 50.4 Gy in 23–28 fractions and 50–66 Gy in 25–33 fractions, 5 fractions per week for nCRT and dCRT, respectively. Standard constraints for organs at risk followed international recommendations. In the period from 2012 to 2017, treatments were based on daily image-guidance with CBCT setup based on a bone match, and from 2017 until 2021, a soft tissue match was performed [15].

### Systemic treatment

Weekly carboplatin (AUC2) and paclitaxel (50 mg/m^2^) were used in the majority of cases. Cisplatin (70–75 mg/m^2^) and 5-Fluouracil (2,100–3,200 mg/m^2^) were an alternative typically used for nCRT followed by surgery for operable patients with resectable cervical tumors. No patients received adjuvant Nivolumab as standard treatment, but the department included few patients in CheckMate 577.

### Surgery

Surgery was performed at earliest 3 weeks after completed nCRT as a joint procedure by two surgeons specialized in upper gastrointestinal surgery and thoracic surgery, with a minimum of 20 esophagectomies per year.

Choice of procedure was left to the surgeon’s discretion. The aim was to achieve a complete resection of the tumor (R0 resection) and a sufficient lymph node dissection. The extent of lymph node dissection depended on the clinical stage (all suspicious lymph nodes during clinical staging should be resected during the surgery) and location of the tumor.

In most cases, an EC resection with a 2- or 3-field lymphadenectomy was performed through a thoracic approach being open, minimally invasive, or a combination of both (hybrid procedure). Depending on the situation, a cervical incision can be indicated. A gastric tube of small bowel was used for reconstruction, in rare cases large bowel was used and in selected cases patients with cervical tumors had laryngectomy and free jejunal graft transplantation [17]. The anastomosis will be located either in the chest or in the neck.

The mandatory (minimal) lymph node dissection will be dependent on the location of the tumor, using the Japanese Classification of Esophageal Cancer (11^th^ Edition): GEJ and distal 1/3 of the esophagus: stations 9 to 13 in the chest and 14 to 18 in the abdomen. Proximal and mid 1/3 of the esophagus: stations 6 to 13 in the chest and 14 to 18 in the abdomen. In case of three-field lymphadenectomy, stations 1 to 5 will be resected in the neck. All suspicious or proven lymph nodes diagnosed during clinical staging to be resected.

### Ethical considerations

Danish law does not require patient consent or notification of the study to the Scientific Ethics Committee. Diagnostic, therapeutic, and follow-up data were obtained from the Danish Esophago-Gastric Cancer database, electronic patient records, and the Eclipse treatment planning system. This study was approved by the Danish Patient Safety Authority (case number 3-3013-2482/1).

### Endpoints

The primary endpoint was OS, and secondary endpoints were loco-regional control, progression-free survival, pattern of failure, and toxicity. Comorbidity was evaluated by the Charlson Co-morbidity Index [18], and PS by the WHO PS criteria. Acute morbidity was defined as an increase in morbidity score within 3 months after treatment, and late morbidity was defined as the highest morbidity score at 3 months or later. No formal scoring or grading of toxicity was done prospectively, and data were retrospectively obtained from the patient files.

### Statistical methods

All analyses were performed in R (version 3.6.1) and SPSS 20. Patient characteristics as well as acute and late toxicity were presented using descriptive statistics. Patient and treatment characteristics were compared by *χ*^2^ tests (categorical variables) and Mann-Whitney *U* tests (continuous variables). All times to events were counted from the RT start date.

Patients were censored at the time of last follow-up, defined as the last contact to the ECV center or death obtained from the electronic patient file. OS was presented as Kaplan-Meier curves, and a multivariable Cox proportional hazards model was fitted for each group to investigate the association between clinical and dosimetric factors and OS. For comparison of OS in different time intervals, patients were divided into three groups based on the year of treatment (2012–2015 vs. 2016–2017 vs. 2018–2021 according to changes in RT technique, see Figure 1). Covariates were selected based on the literature.

Competing risk analysis of first site of failure has divided outcomes into loco-regional failures (LRF), distant metastasis (M), simultaneous LRF+M, and death with no evidence of disease (Death NED). A descriptive comparison of the pattern of failure is shown with stacked cumulative incidence plots comparing dCRT and nCRT as well as adeno carcinoma (AC) and SCC. A *p*-value ≤ 0.05 was considered significant; all tests were two-sided. No correction for multiple testing was done.

## Results

Among the 417 patients identified between 2012 and 2021, 250 patients received nCRT, and 167 patients received dCRT. For nCRT, 59.2% were diagnosed with AC, and 38.4% with SCC, whereas for dCRT, it was 40.7% and 56.9%, respectively. For the entire patient cohort, the median age was 67 years (range 41–88); 78% were male, and the majority of patients had PS 0 or 1 (92%). T3-T4 tumors and node-positive disease were found in 86% and 65%, respectively. Most patients had Charlson comorbidity index of 0 or 1–2 (51% and 40%, respectively).

Patient characteristics according to the planned treatment strategy are presented in Table 1. As expected, patients eligible for nCRT were predominantly male with AC, lower T and N stages, and PS 0–1.

**Table 1:** Patient characteristics.

Variable	nCRT (*n* = 250)	dCRT (*n* = 167)
Gender		
Male	198 (79%)	127 (76%)
Female	52 (21%)	40 (24%)
Histology		
Adenocarcinoma	148 (59%)	68 (41%)
Squamous cell carcinoma	96 (38%)	95 (57%)
Other	6 (3%)	4 (2%)
Performance status		
0–1	234 (94%)	150 (90%)
2+	6 (6%)	17 (10%)
T-stage		
T1–T2	37 (15%)	21 (13%)
T3–T4	213 (85%)	146 (87%)
N-stage		
N0	106 (42%)	44 (26%)
N+	144 (58%)	123 (74%)
Surgery		
yes	231 (92%)	32 (19%)
no	19 (8%)	135 (81%)
Chemotherapy		
Carboplatin/Paclitaxel	224 (90%)	143 (86%)
Cisplatin/5-Fluorouracil	24 (9%)	20 (12.0%)
Other	2 (1%)	2 (1%)
None	0	2 (1%)
Target volumes (median, min-max)		
GTV T	35.9 cm^3^ (4.4–202.0)	37.9 cm^3^ (3.8–245.7)
GTV N	2.8 cm^3^ (0.04–57.3)	3.3 cm^3^ (0.2–89.9)
Total dose prescribed (Gy)		
41.4	193	0
45	23	0
50/50.4 60+	10 0	164 3
Dosimetrics (median, min-max)		
Mean lung dose	8.3 (0.7–16.7) Gy	9.2 (0.3–18.1) Gy
Mean heart dose	15.7 (0.04–29.9) Gy	13.8 (0.06–29.3) Gy

Compliance with RT was high, only 49 patients (12 %) did not complete the prescribed RT dose, and for the nCRT group, 92.4% proceeded to surgery. Reasons for no surgery were patients’ choice, disease progression during nCRT, and poor general condition not applicable for surgery. In total, 53% completed the full prescribed dose of chemotherapy, more commonly for nCRT patients (66%) as compared to dCRT (33%), and another 21% (15% vs. 30%, respectively) needed lowering of the chemotherapy dose.

### Outcome analysis

**Figure 2:**
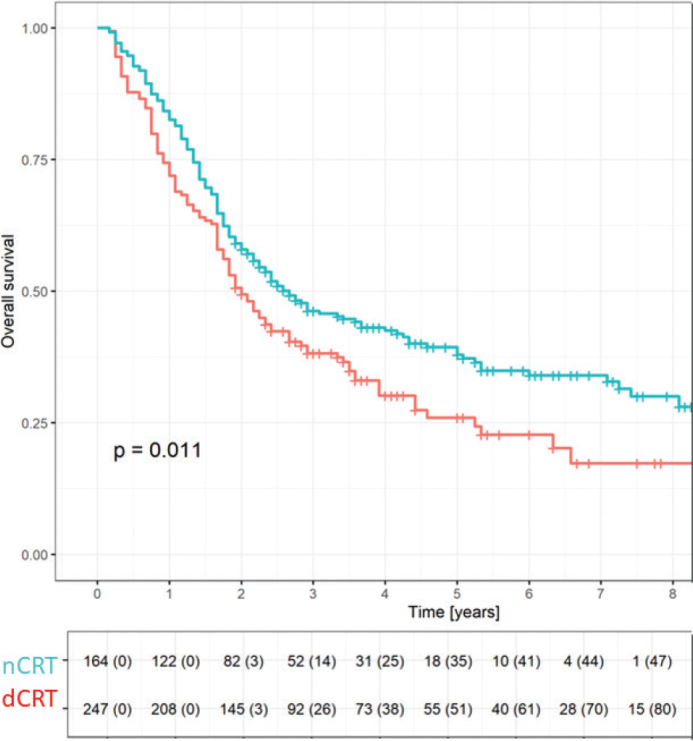
Overall survival for neoadjuvant chemo-radiotherapy (nCRT) and definitive chemo-radiotherapy (dCRT).

Median follow-up for all 417 patients was 24 months with 30% still being alive at the time of analysis. Figure 2 shows the comparison of OS between nCRT and dCRT. As expected, patients treated with nCRT and surgery had a longer median OS of 31 months compared to 24 months for dCRT. The 3-year OS was 46.4% among patients receiving nCRT and surgery and 38.2% among patients treated by dCRT. OS was not affected by time of treatment period (2012–2015 vs. 2016–2017 vs. 2018–2021) in neither nCRT nor dCRT patients.

Multivariable analyses were performed to identify factors associated with OS. For nCRT, worse PS, AC histology, larger GTV N size, and higher mean lung dose (MLD) were significantly associated with poorer OS. For dCRT, worse PS and larger GTV T and GTV N sizes were significantly associated with poorer OS (Table 2).

**Table 2:** Mulitvariate analyses of factors associated with survival in patients treated with neoadjuvant chemo-radiotherapy (nCRT) and definitive chemo-radiotherapy (dCRT) for EC and GEJ cancer, respectively.

Model for survival (HR; *p*-value)	nCRT	dCRT
Performance status PS 0 vs. PS 1	0.5185; *p* = 0.0003*	0.4947; *p* = 0.0025 *
PS 1 vs. PS 2	1.2193; *p* = 0.7131	0.9007; *p* = 0.7662
Histology (SCC vs. AC)	0.6442; *p* = 0.0341 *	0.7595; *p* = 0.2505
Size of GTV T	1.0016; *p* = 0.5724	1.0084; *p* < 0.0001 *
Size of GTV N	1.0347; *p* = 0.0002 *	1.0182; *p* = 0.0386 *
Mean lung dose	1.1049; *p* = 0.0061 *	1.0531; *p* = 0.1767
Mean heart dose	0.9731; *p* = 0.1290	1.0057; *p* = 0.7057
Sex (male vs. female)	0.7977; *p* = 0.3230	0.8644; *p* = 0.5476
Age	1.0206; *p* = 0.0767	1.0138; *p* = 0.2826

HR: Hazard Ratio; AC: adenocarcinoma; SCC: squamous cell carcinoma; GTV: gross tumor volume.

* statistically significant.

**Figure 3:**
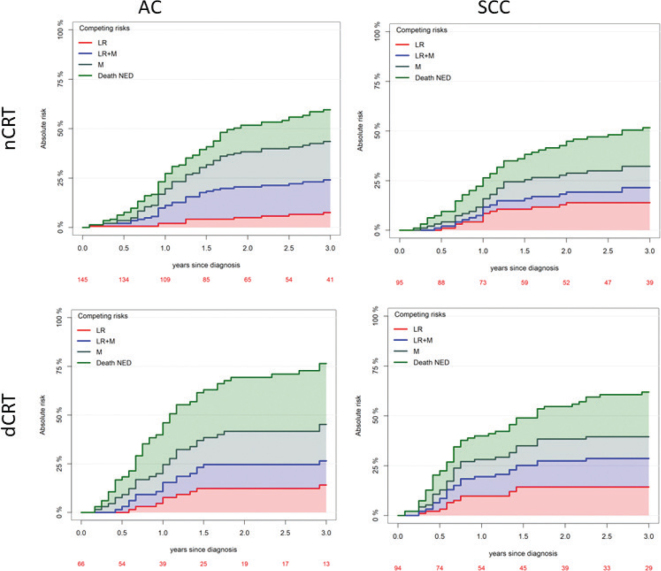
Cumulative incidence of events with competing risk analysis of patients treated with definitive chemo-radiotherapy for squamous cell carcinoma. LR: loco-regional recurrence; LR+M: loco-regional and metastatic recurrence; M: metastatic only recurrence; Death NED: death with no evidence of disease; nCRT: neoadjuvant chemo-radiotherapy; dCRT: definitive chemo-radiotherapy; AC: adenocarcinoma; SCC: squamous cell carcinoma.

Among all 417 cases, recurrent disease occurred in 42%, of which 10% had oligometastatic disease. Median time to recurrence was 20 months. In this study, the four treatment groups nCRT AC, nCRT SCC, dCRT AC, and dCRT SCC showed different patterns of failure (Figure 3). In general, AC patients more often experienced metastatic failure only, and the risk of death without evidence of disease was highest for dCRT AC patients (Figure 3). In nCRT, the frequency of LRFs only was higher for SCC, but in general, this group experienced the lowest risk for recurrence or death NED.

### Toxicity

Ninety-six percent of patients experienced some kind of acute toxicity during or immediately after treatment, more common in dCRT patients compared to nCRT patients (Table 3). The most common side effects were pain, dysphagia/esophagitis, and fatigue. Side effects were generally mild and manageable, but 1/3 required hospitalization, and one patient died due to grade 5 acute toxicity (infection). Late side effects were not systematically recorded; the most common were pain, fatigue, and dysphagia, with 12% treated with dilatation and 7% receiving a stent. No data on surgical complications were collected.

**Table 3:** Acute and late toxicity in neoadjuvant chemo-radiotherapy (nCRT) and definitive chemo-radiotherapy (dCRT), respectively.

	nCRT (*n* = 250)	dCRT (*n* = 167)
**Acute toxicity**		
Diarrhea	11 (4%)	14 (8%)
Obstipation	66 (27%)	42 (25%)
Nausea and vomiting	66 (267%)	54 (33%)
Febrile neutropenia	3 (1%)	12 (7%)
Dysphagia	106 (43%)	86 (52%)
Pain	165 (67%)	125 (75%)
Sensory neuropathy	0 (0%)	3 (2%)
Fatique	91 (37%)	76 (46%)
Pneumonitis	1 (0.4%)	1 (0.6%)
Esophagitis	15 (6.0%)	15 (9%)
Infection	33 (13%)	42 (25%)
Other	89 (36%)	96 (58%)
**Late toxicity**		
Dysphagia	4 (2%)	4 (2%)
Pain	2 (0.8%)	2 (1%)
Fatique	0 (0%)	2 (0.2%)
Dyspnea	4 (2%)	4 (2%)
Esophageal stenosis	19 (8%)	29 (18%)
Esophageal fistula	3 (1%)	1 (0.6%)
Ulceration	0 (0%)	0 (0%)
Other	20 (8%)	17 (10%)

## Discussion and conclusion

In this study, we examined survival and morbidity following concurrent CRT in patients with EC or GEC treated through a 10-year period in a single institution. This study showed a median OS of 31 versus 24 months and a 3-year OS of 46.4% versus 38.2% for nCRT and dCRT, respectively. This is in line with previously published data [10, 13, 19, 20].

Radiotherapy outcome depends highly on both chemotherapy, RT, and surgery as well as histology (AC vs. SCC). As expected, patients receiving nCRT in the current study had better outcomes compared to patients treated with dCRT. Patients offered surgery are usually diagnosed with less comorbidity, better PS, and smaller tumors, thus receiving RT to smaller treatment volumes. Our data are in line with data from Jeon et al. [18], who published a retrospective study of stages II and III EC patients (both AC and SCC) comparing outcomes after nCRT, dCRT, RT alone, and perioperative chemotherapy. Data showed improved OS for nCRT compared to dCRT or RT alone, whereas perioperative chemotherapy was associated with improved OS but only for AC. A recent real-world data analysis of patients in the Netherlands with EC and GEJ AC [17] compared outcomes of neoadjuvant CRT using the CROSS regimen to perioperative chemotherapy outcomes. They reported a median OS of 33.7 months (95% confidence interval [CI] 32.0–35.6), with a 3-year OS rate of 48.1% in the CROSS arm. In comparison, the Neo-Aegis and ESOPEC trials reported 49.2 months and 37 months OS, respectively, as well as 57% and 50.7% 3-years OS, respectively, in the CROSS arms [8, 9]. Our outcome data are in line with the Dutch real-world data but lower than trial data from CROSS and Neo-Aegis. This is expected since patients included in trials usually have better PS (no PS 2 allowed), smaller stages, and less comorbidity.

Knowledge of risk factors associated with treatment outcome is essential to select the optimal treatment for the individual patient. In this study, we found worse PS, AC histology, large GTV N size, and high MLD significantly associated with poorer OS for patients receiving nCRT. This is in line with previously published data [6, 21–23]. The CROSS study showed survival gain from nCRT in both AC and SCC, but median OS was superior in SCC [6]. Nam et al. reported advanced stage, poor PS, and poor treatment response as independent factors for decreased survival [20] but did not examine dosimetric factors. The correlation to RT dose has been examined in other studies, though. Lin et al. [19] found lung V20Gy and V5Gy were associated with decreased OS but not mean heart dose, whereas Xu et al. [21] found that heart V30Gy >45% and MLD >10 Gy were independently associated with worse survival after adjustment for other clinical and dosimetric factors in a cohort of 560 patients treated with CRT with or without surgery for EC. Heart and lung doses were also found to be risk factors for radiation-induced cardiac and pulmonary complications (*P* < 0.05), which were accordingly associated with worse survival. In a subgroup analysis of patients in the surgery cohort, heart V30Gy remained independently associated with OS after adjusting other variables (*P* = 0.001), while in the non-surgery cohort, MLD remained independently associated with OS after adjusting for other variables (*P* = 0.028). We found MLD important for OS as well, but no association with mean heart dose was found. Xu et al. analyzed the variables mean heart dose >27 Gy and V30Gy>45%, which are considerably higher than our mean heart doses of 15.7 Gy and 13.8 Gy for nCRT and dCRT, respectively, and might explain the difference.

In the present study, for patients receiving dCRT, worse PS and large GTV T and GTV N sizes were significantly associated with OS, but no correlation with dosimetric factors was identified. The dCRT group included more patients with SCC (57%) compared to the nCRT group (SCC = 38%), which reflects clinical practice and could explain some of the difference in risk factors in the final models. The correlation between OS and PS and target volume is in line with other studies [24, 25], indicating that both patient-related and disease-related factors are important. More patients in the dCRT group experienced loco-regional recurrence or death without evidence of disease; perhaps implying that loco-regional recurrence reflects target volume response – more likely in smaller tumors – whereas death without evidence of disease reflects the general health state of the patient (PS).

Data on recurrence patterns after CRT in EC and GEJ cancer are sparse. In our data, we found that the most common recurrence site for SCC was loco-regional recurrence, whereas the most common recurrence for AC included a metastatic site. This is in line with the findings of Kim et al. [26] but contrary to the findings of Smit et al. [27]. Interestingly, we saw a larger proportion of patients dying without evidence of disease in all treatment groups, except AC treated with nCRT. For dCRT patients, this might be explained by comorbidity and poorer general health state, but death from cancer is possible as well if the patient’s general health did not allow for treatment. No clear explanation exists for SCC treated with nCRT. More data on patterns of failure are needed to help select the optimal treatment for these patients. The outcome of the Neo-Aegis trial stated that there was no significant difference in loco-regional alone versus metastatic disease alone versus combined recurrence between treatment groups. No data on pattern of failure were specified in the ESOPEC study.

Limitations of the current study include its retrospective nature including the lack of data on surgical procedures, complications to surgery as well as no prospective scoring of toxicity. Some strengths may be noticed as well. In Denmark, all healthcare treatments are equally available to all patients due to public financing, and data on follow-up and death are comprehensive due to the personal ID number given to all Danish citizens by birth and a joint electronic patient file. Furthermore, patients in this study were treated with modern and well-described state-of-the-art RT. The added value of this study is the reporting of real-world outcomes for patients with a diagnosis where treatment strategies are changing. To interpret the impact of any treatment on unrestricted patient groups, knowledge of the implementation and outcomes of previous treatments is crucial. This includes knowledge of patterns of failure and risk factors associated with patient outcomes.

In conclusion, OS among EC patients treated with nCRT or dCRT in our clinical practice is in line with results from published literature. There was a high adherence to treatment strategy as the majority of patients treated with nCRT went to surgery. Treatment side effects to nCRT and dCRT are frequent but largely manageable.

## Supplementary Material



## Data Availability

The data of this study can be accessed by direct contact to the corresponding author by mail.
