# Supplementary material for: Real-world outcomes after concurrent chemo-radiotherapy in patients with locally advanced esophageal and gastroesophageal junction cancer
Source: Acta Oncol. 2025 Oct 15;64:44013. doi: 10.2340/1651-226X.2025.44013 (PMC12534993; doi:10.2340/1651-226X.2025.44013)
Supplement: Supplementary file 1 [file AO-64-44013-s1.pdf]

Supplementary material has been published as submitted. It has not been copyedited, or typeset by Acta Oncologica

National guidelines for radiotherapy in esophageal, gastro-esophageal junction

Danish esophageal, gastroesophageal junction cancer and stomach cancergroup (DECV)

ABBREVIATIONS

BSA	Body surface area
CI	Confidence interval
CT	Computed tomography
CTV	Clinical target volume
CTVt	Clinical target volume tumor
CTVn	Clinical target volume lymph nodes
CTV elective	Clinical target volume elective
CTV total	Clinical target volume tumor + clinical target volume lymph nodes + elective volumina
CTCAE	Common terminology criteria for adverse events
DCCC	Danish Comprehensive Cancer Center
DCPT	Danish Center of Particle therapy
DECV	Danish esophagus, gastroesophageal junction cancer and ventricular cancer group
DFS	Disease-free survival
DMCG	Danish Multidisciplinary Cancer Group
EBUS	Endobronchial ultrasound
ECG	Electrocardiogram
ECV	Esophagus, gastroesophageal junction cancer and ventricular cancer
eCRF	Electronic case report form
ESMO	European Society for Medical Oncology
ESTRO	European Society for RadioTherapy and Oncology
EUS	Endoskopic ultrasound
FDG	Fluorodeoxyglucose
GEJ	Gastro esophageal junction
GTV	Gross tumor volume
GTVt	GTV tumor
GTVn	GTV nodes
ICD	International classification of diseases
IRB	Institutional review board
ITT	Intention to treat
ITV	Internal tumor volume
MDT	Multidisciplinary team
NYHA	New York Heart Association
OS	Overall survival
PET	Positron emission tomography
PFS	Progression-free survival
PRV	Planning organ at risk volume
PTV	Planning target volume = CTV + ITV + set-up margin (varies with center depending on fixation, strategy for daily match and adaptation).
RT	Radiotherapy

Epidemiology

Oesophageal and gastroesophageal junction (GEJ) cancer is the eighth most common cancer worldwide, with an estimated 456,000 new cases in 2012 (3.2% of the total), and the sixth most common cause of death from cancer with an estimated 400,000 deaths (4.9% of the total).

Tumors in the esophageal tube are predominantly sqaumous cell carcinomas in the upper esophagus and adenocarcinomas in the distal esophagus. Tumors in Gastro - Esophageal Junction (GEJ) are predominantly adenocarcinomas.

Esophageal cancer is anatomically located cervical or high, middle or low thoracic, respectively. Cardia cancer is anatomically located in the GEJ corresponding to the gastroesophageal transition.

Regional metastatic lymph nodes should be verified by biopsy and/or defined evident metastatic on Ultrasound, CT or PET/CT imaging. Supraclavicular and infradiagfragmatic lymph nodes are recommended to be verified by biopsy unless M1 disease is obvious on imaging.

Diagnostics and multidisciplinary teams (MDT)

Treatment of oesophageal and GEJ cancer is anchored in the multidisciplinary teams, discussing and advising the choice of treatment based on prognosis, TNM stage of the cancer, the possibility of resection (resectability) and patient's general state (operability).

Primary diagnostic investigation includes gastroscopy and biopsy-proven histological diagnosis as well as PET / CT with iv. contrast. Lymph nodes with FDG uptake are considered malignant. EUS and/or EBUS as well as MR and/or ultrasound-guided lymph node biopsy are optional. Volumes with FDG uptake and suspects of malignancy should be biopsied after recommendation from the MDT or a specialist in nuclear medicine.

2.TMN classification and stage

TNM classification and stage follows UICC TNM classification, version 8.

"Rules for Classification" states that tumors in the esophagogastric transition (OGJ) whose epicenter is within the oral 2 cm of cardia (Siewert type I/II) should be staged as esophageal cancer. Tumors whose epicenter is more than 2 cm anal for OGJ should be staged as ventricular cancer.

3. TREATMENT WITH CURATIVE INTENT

Operation alone

- Patients in PS 0-1 with T1 tumor (rarely T2) N0 M0 is offered surgery without oncological neoadjuvant treatment if the patient is assessed operable and tumor assessed resectable.
- No oncological adjuvant treatment if surgery is microradically removed (R0 resection)

Chemo-Radiotherapy (chemo-RT) followed by surgery

- Patients in PS 0-1 and assessed operable, with loco-regional squamous cell carcinoma or adenocarcinoma, and cT1-3 cN0-3 M0, who are assessed resectable at MDT conference.
- Perioperative chemotherapy is an equivalent alternative to preoperative chemo-RT for adenocarcinomas in distal GEJ and ventricular involvement.

Radiotherapy dose:

50 Gy/25-28 F, 5 F/W concomitant with Cisplatin/5-FU or Carboplatin/Paclitaxel (modified CROSS)

or

41,4 Gy/23 F, 5 F/W concomitant with Carboplatin/Paclitaxel (nCROSS).

5. TREATMENT PLANNING AND DELIVERY

Treatment start

Radiotherapy initiation concomitant with chemotherapy is recommended following cancer patient pathways and national recommended course times of diagnostic workup and treatment start.

Treatment technique

Markers: At the time of diagnostic endoscopy, markers can be applied orally and anal in the primary tumor to be used for target definition, and for assessing target position in daily treatments.

Position and fixation: The patient is treated with arms fixed above the head (high, middle, low esophageal cancer) or arms down the side (cervical esophageal cancer).

Planning CT scan: A 4D-CT is used for radiotherapy planning. 4D-CT scans are used to assess tumor movements and for delineation of the GTV using mid-ventilation, mid-position, Maximum Intensity Projection (MIP) or similar methods [Underberg 2005, Wolthaus 2006]. PET-CT is recommended for target definition and gating techniques like eg. Deep Inspiration Breath Hold (DIBH) can be used for daily treatments. Use of i.v.-contrast is mandatory and CT slices in tumor areas should be 3 mm maximum.

Radiotherapy planning: Advanced 3D-CT based planning techniques are used like IMRT or VMAT, with a modern dose algorithm (eg Monte Carlo (MC), AAA, Collapsed Cone (CC) or equivalent). Multiple fields or arc techniques, co-planar or non-coplanar techniques may be used, but direct opposing fields should be avoided.

Target coverage: GTV and CTV should be covered by the 95% isodose curve (99% of the volume). PTV should be covered by the 95% isodose curve if possible, especially in mediastinum. Every slice should be reviewed to ensure target coverage.

IGRT: Margins for PTV should be supported by an image guidance protocol. Daily image guidance with soft tissue match is recommended with regular evaluation for treatment adaptation.

Energy: Photons 6-10 MV.

Target definition

- **GTV:** Tumor + primary involved lymph nodes based on histology and PET-CT scans. Tumor and lymph nodes in mediastinum is delineated in a ”mediastinal window”.

- **GTVt:** Primary tumor. Delineated based on endoscopy, PET/CT and EUS if performed.

- **GTVn:** Patological lymph nodes. Delineated based on endoscopy, EUS if performed, PET/CT, ultrasound and MR.

- **CTVt:** *Esophageal and GEJ tumors*: CTVt = GTVt + oesophageal ”fat-pad” 3 cm cranio-caudally for GTVt. When delineating the oesophageal ”fat-pad” v. azygos is included. In relation to aorta, the "fat-pad" is included in half the aorta circumference radially and posteriorly. With tumor growth into the trachea or thyroid, these structures are included. For tumors in the lower esophagus and GEJ, CTV can be restricted to 2 cm distal to tumor but should include a. gastrica sinistra. For tumors in cervical esophagus, CTV may be restricted proximally, with upper border being the larynx. The CTV should be shaped for large vessels, bones and lungs, unless there is tumor growth into these organs.

  - **CTVn:** *Lymph nodes*: CTVn = GTVn + the involved lymph node region, however at least 1 cm proximally and distally. Large vessels, bones, heart, trachea and lungs are included only if direct tumor growth into these organs.

  - **CTV elective:** If the primary tumor is located above the carina with lymph node metastases the periclavicular lymph node regions are delineated bilaterally. Radial border is the clavicles. For tumors without lymph node metastases, elective lymph node regions are only delineated in T3/T4 tumors. Muscles and bones are not included.

    In definitive radiotherapy, lymph nodes deemed suspicious for malignancy on CT is included whenever possible.

    Mediastinum: Distal to v. brachiocephalica sin. this vessel is the anterior border. Proximal to v. brachiocephalica sin. mediastinum anterius is included to the sternum.

  - **CTVtotal: CTVt + CTVn.** CTV total is the target for treatment planning. If CTV-t and CTV-n are far apart, these two volumes can be used for treatment planning.

  - **PTV:** CTV + institutional margin.
